# Causal interaction inference of compensatory structures from single-cell perturb-seq in AML

**DOI:** 10.1093/bib/bbaf631.007

**Published:** 2025-12-12

**Authors:** Changde Cheng, Sajesan Aryal, Brittany M Curtiss, Xinyue Zhou, Rui Lu

**Affiliations:** Department of Medicine, Division of Hematology/Oncology, University of Alabama at Birmingham, Birmingham, AL, United States; O’Neal Comprehensive Cancer Center, University of Alabama at Birmingham, Birmingham, AL, United States; Department of Biomedical Informatics and Data Science, University of Alabama at Birmingham, Birmingham, AL, United States; Institute for Cancer Outcomes and Survivorship, University of Alabama at Birmingham, Birmingham, AL, United States; Department of Medicine, Division of Hematology/Oncology, University of Alabama at Birmingham, Birmingham, AL, United States; O’Neal Comprehensive Cancer Center, University of Alabama at Birmingham, Birmingham, AL, United States; Department of Medicine, Division of Hematology/Oncology, University of Alabama at Birmingham, Birmingham, AL, United States; O’Neal Comprehensive Cancer Center, University of Alabama at Birmingham, Birmingham, AL, United States; Department of Medicine, Division of Hematology/Oncology, University of Alabama at Birmingham, Birmingham, AL, United States; O’Neal Comprehensive Cancer Center, University of Alabama at Birmingham, Birmingham, AL, United States; Department of Medicine, Division of Hematology/Oncology, University of Alabama at Birmingham, Birmingham, AL, United States; O’Neal Comprehensive Cancer Center, University of Alabama at Birmingham, Birmingham, AL, United States

## Abstract

Resistance to single-agent therapy in acute myeloid leukemia (AML) often arises from compensatory epigenetic circuits. We present a causal inference framework that treats therapy as an intervention (do-operator) and defines resistance as the deviation of dual perturbations from the additive expectation of monotherapies. By modeling the causal structure of the leukemia stem-cell epigenetic landscape that maintains identity and fitness, the framework shows that synergistic and antagonistic interactions can be inferred directly from single perturbations, suggesting interaction inference may be simpler than previously assumed. We applied this model to Perturb-seq of 16 epigenetic regulators in KMT2A-rearranged AML (>31,000 single-cell transcriptomes). From single-knockout profiles, the framework correctly predicted synergistic pairs (Menin+KAT6A, Menin+DOT1L) that enhanced differentiation and induced cell death, as well as an antagonistic pair (DOT1L + PCGF1) that conferred resistance. Predictions were validated by pharmacologic inhibition and bulk RNA-seq, confirming the model’s predictive accuracy. This work presents a mechanism-guided approach for rational prioritization of epigenetic drug combinations in AML and other cancers.

